# IPO9 Promotes Ovarian Cancer Progression by Suppressing HMOX1‐Dependent Ferroptosis

**DOI:** 10.1155/humu/8545131

**Published:** 2026-01-21

**Authors:** Yimei Meng, Peiling Li

**Affiliations:** ^1^ Department of Obstetrics and Gynecology, The Second Affiliated Hospital of Harbin Medical University, Harbin, Heilongjiang, China, hrbmush.edu.cn

**Keywords:** ferroptosis, HMOX-1, immune microenvironment, IPO9, ovarian cancer

## Abstract

Ovarian cancer (OC) poses a significant threat to women’s health, with current treatment strategies remaining suboptimal, necessitating the exploration of novel therapeutic targets and immune microenvironment dynamics. This study integrates multiomics data from TCGA, GEO, and IEU‐Open‐GWAS, employing scRNA‐seq, scPagwas, BayesPrism, and WGCNA to identify key cell subpopulations and genes, followed by functional validation through EdU, colony formation, Transwell assays, and ferroptosis markers (MDA, ROS, and ferrous ions). Results reveal MALAT1^+^ epithelial cells as a core cell subpopulation in OC, with higher abundance correlating with shorter overall survival, suppressed immune microenvironments, and potential immunotherapy resistance, while their infiltration levels are closely associated with OC immune dynamics and somatic mutations. Further analysis identifies *IPO9* as a core gene upregulated in OC, promoting tumor progression by inhibiting HMOX1‐dependent ferroptosis. These findings highlight MALAT1^+^ epithelial cells as drivers of immune suppression in OC and propose IPO9 as a promising therapeutic target, offering new avenues for immunotherapy development.

## 1. Introduction

Ovarian cancer (OC) is acknowledged as one of the most fatal gynecological malignancies, exhibiting high mortality and substantial limitations in early detection and treatment. Patients diagnosed at advanced stages have a low 5‐year survival rate [1]. Established risk determinants for OC encompass advancing age, familial inheritance patterns, germline susceptibility loci (such as *BRCA* gene variants), reproductive characteristics (nulliparity and late menopause), and modifiable factors (obesity, sedentary behavior, and tobacco consumption) [2, 3]. From a clinical manifestation perspective, OC frequently manifests insidiously through nonspecific abdominal discomfort, pelvic pressure sensations, gastrointestinal distension, micturition pattern alterations, and menstrual cycle abnormalities. This diagnostic ambiguity frequently results in postponed clinical recognition and frequent identification during metastatic disease phases [4]. Management of OC generally involves a multimodal strategy integrating surgery, chemotherapy, targeted therapy, and immunotherapy to enhance treatment efficacy and optimize prognosis [5]. However, high recurrence rates and therapeutic resistance persist, leaving patient outcomes unsatisfactory [6, 7]. Thus, deeper insights into OC pathogenesis and novel therapeutic/diagnostic targets are urgently needed.

Tumor heterogeneity denotes the presence of distinct cell subpopulations within a tumor, each characterized by unique genetic, phenotypic, and functional attributes. This cellular diversity is a critical pathological feature that influences tumor biology, treatment responsiveness, and clinical prognosis [8]. In OC, heterogeneity is complex and multidimensional; it is evident both between patients (intertumor heterogeneity) and within individual tumors (intratumor heterogeneity), resulting in diverse cellular behaviors and varied therapeutic responses. From the perspective of cell subpopulations, OC heterogeneity is manifested by the coexistence of malignant cell subtypes with distinct molecular profiles. Single‐cell transcriptomic analyses have demonstrated that specific subpopulations, such as tumor cells expressing biomarkers like CA125 (MUC16) and tumor‐initiating cells with stem cell properties, are significantly associated with poor prognosis [9]. At the molecular level, heterogeneity arises from the spatiotemporal variation in genomic alterations, epigenetic remodeling, and metabolic reprogramming. For example, in high‐grade serous ovarian cancer (HGSOC), intratumor heterogeneity is reflected by distinct transcriptomic profiles among various cell subpopulations, which may drive the emergence of chemotherapy‐resistant phenotypes via the activation of stem cell‐associated pathways such as Wnt/*β*‐catenin [10, 11]. Additionally, in ovarian clear cell carcinoma, cancer cells activate cancer‐associated fibroblasts (CAFs) via the platelet‐derived growth factor (PDGF) signaling pathway; the activated CAFs subsequently induce feedback activation of HIF‐1*α* in cancer cells through metabolic reprogramming, thereby establishing a positive feedback loop that drives tumor progression [12]. In view of this multilayered heterogeneity, conventional analytical methods based on bulk tumor samples exhibit significant limitations. Emerging precision medicine strategies now emphasize the use of technologies like single‐cell sequencing to delineate the composition of tumor cell subpopulations and incorporate specific biomarkers (e.g., *BRCA* mutation status) into individualized treatment plans. Clinical studies have demonstrated that targeted therapies such as PARP inhibitors can substantially extend progression‐free survival (PFS) in patients with defined molecular subtypes [10, 13]. Consequently, an in‐depth analysis of the spatiotemporal evolution of tumor heterogeneity and the development of combination therapies that target key subpopulations and their interactions with the microenvironment represent critical research directions for improving OC treatment efficacy. Critically, among OC cell populations, epithelial cells—key drivers of tumor progression—have not been systematically characterized for their subtype‐specific roles in immunosuppression and therapeutic resistance, which is a critical gap hindering targeted therapy development.

**Figure 1:**
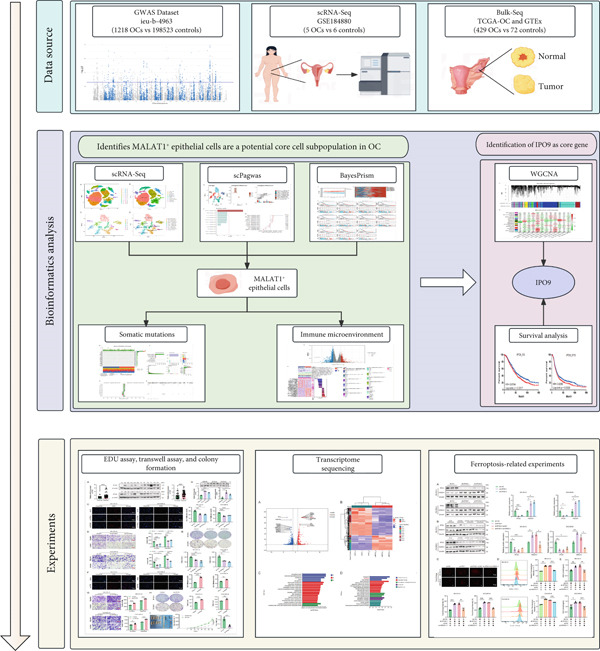
Flowchart of this study.

To address the aforementioned gaps in understanding OC heterogeneity and its interplay with the immune microenvironment, this investigation employs an integrative approach combining single‐cell RNA sequencing (scRNA‐seq) datasets, genome‐wide association study (GWAS) information, and bulk tissue expression profiles to systematically characterize pivotal epithelial cellular clusters in OC through multidimensional omics analyses. The research further examines their clinical correlations with survival outcomes, immunomodulatory niche characteristics, and genomic mutation patterns. Key molecular markers linked to these cellular subgroups are subsequently identified and experimentally validated, offering novel conceptual frameworks and candidate therapeutic targets for OC management. The experimental workflow is comprehensively outlined in Figure 1.

## 2. Materials and Methods

### 2.1. Cell Culture and Tissue Samples

OC cell line SK‐OV‐3 (RRID: CVCL_0C84) and normal ovarian epithelial cell line IOSE‐80 (RRID: CVCL_5546) were purchased from Shanghai Zhongqiao Xinzhou Biotechnology Co., Ltd. (Shanghai, China), whereas OC cell line OVCAR‐8 (RRID: CVCL_1629) was obtained from Beijing Zhongsheng Aobo Biotechnology Co., Ltd. In addition, the HEK‐293T cell line was provided by Abcell. SK‐OV‐3 was cultured in RPMI‐1640 medium (Gibco, United States) supplemented with 1% penicillin/streptomycin (Beyotime, Shanghai, China) and 10% fetal bovine serum (Procell, Wuhan, China), whereas IOSE‐80, HEK‐293T, and OVCAR‐8 were maintained in DMEM (Gibco, United States) containing 1% penicillin/streptomycin (Beyotime, Shanghai, China) and 10% fetal bovine serum (Procell, Wuhan, China). All cell lines were incubated at 37°C in a humidified atmosphere with 5% CO_2_.

Normal ovarian tissue samples (*n* = 48) and OC tumor tissue samples (*n* = 48) were collected from hospitalized patients at the Second Affiliated Hospital of Harbin Medical University. The clinical characteristics of 48 OC samples are listed in Table S1. The experimental protocol was approved by the Medical Ethics Committee of the Second Affiliated Hospital of Harbin Medical University (Approval No. Ky2020‐060), and written informed consent was obtained from all participants.

### 2.2. Transfection

Plasmids used in this study were obtained from Synbio Technologies (Suzhou, China). For lentiviral packaging, the pXPax2 and pMD2.G plasmids were utilized. The pXPax2 plasmid encodes essential components for viral packaging, while the pMD2.G plasmid provides the vesicular stomatitis virus glycoprotein (VSV‐G) envelope for pseudotyping. These plasmids were cotransfected with the target transfer plasmid into HEK293T cells to generate lentiviral particles. Following lentiviral transduction, target cells were selected using puromycin. The optimal puromycin concentration was determined through a kill curve assay to ensure the complete elimination of untransduced cells. Transduced cells were cultured in medium containing puromycin for 7–14 days to establish stable polyclonal populations. Successful transgene integration was verified by quantitative real‐time PCR (qRT‐PCR) analysis.

To inhibit endogenous HMOX1 expression, two siRNAs targeting *HMOX1* were designed and synthesized by Sangon (Shanghai, China). When the cells reached 50%–60% confluency, they were transfected with *HMOX1* siRNAs using CALNPTM RNAi transfection reagent (D‐nano Therapeutics, Beijing, China). The siRNA‐transfected cells were harvested at 24 or 48 h posttransfection for functional studies. Table S2 enumerates the siRNA sequences.

### 2.3. qRT‐PCR

Cellular and tissue RNA isolation was conducted following the manufacturer’s protocol with TRIzol reagent (Invitrogen, United States). The obtained RNA was then converted to cDNA through reverse transcription employing the TOYOBO reverse transcription system (Osaka, Japan). Amplification reactions were executed on a LightCycler 96 instrument (Roche, Germany) utilizing ChamQ SYBR qPCR Master Mix (Vazyme, Nanjing, China). Sample standardization against *18s rRNA* served as an endogenous reference. Primer synthesis was commercially obtained from Sangon Biotech (Shanghai, China). Transcript abundance of *IPO9* and *HMOX1* was quantified through the 2^−*ΔΔ*CT^ method relative to *18s rRNA*. Table S3 enumerates the primer sequences.

### 2.4. Western Blot

Protein expression profiles in human tissue specimens and ovarian‐derived cell models were assessed through lysate preparation employing RIPA buffer, with subsequent concentration determination via NanoDrop One spectrophotometric analysis (Thermo Fisher, United States). Electrophoretic separation was conducted using 10% SDS‐PAGE followed by transference to nitrocellulose membranes (Pall, Port Washington, New York, United States). Membranes underwent 1‐h ambient temperature blocking prior to 16‐h incubation at 4°C with primary antibodies targeting IPO9 (Zenbio, 861793), *β*‐actin (Proteintech, 66009‐1‐Ig), and HMOX‐1 (Zenbio, R24541). Detection was achieved through sequential treatment with HRP‐conjugated secondary antibodies (Zenbio, Chengdu, China) for 60 min and chemiluminescent signal development using an ECL system (Beyotime, Shanghai, China).

### 2.5. Cell Growth and Proliferation Assay

The proliferation capacity of cells was assessed using the EdU Cell Proliferation Detection Kit (Beyotime, Shanghai, China; Cat# C0078S) following standardized protocols. Seeding in 24‐well plates occurred prior to culture under optimal growth conditions. Cells underwent 2‐h incubation with EdU solution at 10 *μ*M concentration before sequential processing: fixation with 4% paraformaldehyde, permeabilization using 0.3% Triton X‐100, and 15‐min exposure to click reaction mixture. Nuclei were counterstained with Hoechst 33342 followed by microscopic documentation (Olympus, Japan).

In parallel colony formation experiments, OVCAR‐8 cells (1 × 10^3^) and SK‐OV‐3 cells (1.5 × 10^3^) were plated in 6‐well plates with medium renewal triweekly. Post 14‐day incubation, cellular specimens underwent fixation with 4% paraformaldehyde and 20‐min staining with 0.1% crystal violet (Solarbio, Beijing, China). Following PBS washes, colonies exceeding 50 cellular units were quantified.

### 2.6. Transwell Migration and Invasion Assay

Cell migratory capacity was evaluated employing 24‐well Transwell inserts (Corning, New York, United States; #3422). Suspensions containing 3 × 10^4^ cells in 0.2 mL serum‐free medium were placed in upper compartments, with 0.6 mL medium supplemented with 10% FBS filling lower chambers. Post 36‐h incubation, cellular specimens underwent sequential processing: fixation with 4% paraformaldehyde, 0.1% crystal violet staining, PBS rinsing, and photodocumentation. Invasion analysis incorporated Matrigel‐coated inserts (Corning, New York, United States; #356234) in identical chambers. Quantitative assessment of translocated cells was performed using 10× microscopic field enumeration.

### 2.7. Reactive Oxygen Species (ROS) Detection

The measurement of intracellular ROS was conducted utilizing the ROS Detection Kit (Beyotime, Shanghai, China; Cat# S0033S). Following trypsin‐mediated dissociation and collection, cellular suspensions were subjected to 30‐min incubation with 10 *μ*M DCFH‐DA at 37°C. Postincubation washing procedures involved three cycles of serum‐free medium treatment and centrifugation. Pelleted cells were reconstituted in 0.5 mL PBS for fluorescence intensity quantification via DxFLEX flow cytometer (Beckman Coulter, United States). FlowJo analytical software was employed for data interpretation.

### 2.8. Ferrous Ion (Fe2^+^) Detection

Cell cultures were established in 24‐well plates with overnight incubation under standard conditions (37°C, 5% CO_2_). Following medium aspiration, three sequential rinses using serum‐free solution were performed. Cellular systems were then treated with FerroOrange working solution (1 *μ*mol/L; DOJINDO, Japan, F347) under identical incubation parameters. Fluorescence microscopy imaging was immediately conducted poststaining without additional washing procedures.

### 2.9. Malondialdehyde (MDA) Detection

Relative intracellular MDA content was quantified using the Lipid Peroxidation Detection Kit (Beyotime, Shanghai, China; Cat# S0131S) in accordance with the manufacturer’s protocol. Cell or tissue samples were lysed with RIPA lysis buffer (Cat# P0013B, Beyotime, Shanghai, China) and centrifuged at 12,000 g for 10 min. Protein concentration was measured using a NanoDrop One spectrophotometer. Subsequently, 100 *μ*L of the experimental supernatant was mixed with 200 *μ*L of the MDA detection reagent and heated at 100°C for 15 min. After thermal processing, samples were allowed to equilibrate to room temperature prior to measuring absorbance at 532 nm with a microplate reader.

### 2.10. OC Xenograft Animal Model

Female BALB/c nude mice (4–6 weeks old, specific pathogen‐free [SPF] grade) were obtained from Liaoning Changsheng Biotechnology Co., Ltd. (Shenyang, China) and housed under pathogen‐free conditions at the Animal Experiment Center of Harbin Medical University. All experimental procedures followed national animal care guidelines and were approved by the Animal Ethics Committee of the Second Affiliated Hospital of Harbin Medical University (Approval No. YJSDW2023‐108). OC xenograft models were generated through subcutaneous implantation of SK‐OV‐3 cells (shNC or sh‐IPO9‐1 variants) into female BALB/c nude mice. Each animal received 5 × 10^6^ cells via dorsal subcutaneous injection, with experimental cohorts comprising five mice per group. Tumor volume was assessed every 3 days. Post 28‐day observation period, anesthetic administration was performed using 250 mg/kg Avertin (i.p.; MilliporeSigma, Burlington, Massachusetts, United States) prior to euthanasia via cervical dislocation. Resected tumor specimens underwent photographic documentation and gravimetric analysis. All procedures were conducted at Harbin Medical University’s Animal Experiment Center under SPF containment protocols.

### 2.11. Transcriptome Sequencing and Analysis

To comprehensively characterize genome‐wide transcriptional alterations resulting from IPO9 suppression, we implemented RNA sequencing–based transcriptomic profiling. Total RNA isolation was conducted in triplicate from both control and IPO9‐knockdown cellular samples using TRIzol reagent (Invitrogen). RNA quality validation was achieved through Agilent 2100 Bioanalyzer assessment (RIN ≥ 8.0). Directional cDNA library preparation was performed with NEBNext Ultra II Directional RNA Library Prep Kit (Illumina), adhering strictly to manufacturer specifications. High‐resolution sequencing (150 bp paired‐end) was executed on the NovaSeq 6000 platform (Illumina), generating ~40 million high‐quality reads per biological replicate.

Primary sequence data underwent preprocessing via TrimGalore (v0.6.7) for adapter removal and quality control, followed by genome alignment against GRCh38.p13 reference using STAR (v2.7.9a) with default parameters. Transcript abundance estimation was calculated through featureCounts (v2.0.3) with GENCODE v44 annotations. Differential gene expression determination employed DESeq2 (v1.38.3) with significance criteria: adjusted *p* value < 0.05 and absolute log2 fold change > 1. Pathway enrichment investigations incorporated clusterProfiler (v4.8.3), utilizing GO and KEGG databases for functional annotation.

### 2.12. Bioinformatics Analysis

The dataset with accession number GSE184880 was obtained from the Gene Expression Omnibus (GEO) database (https://www.ncbi.nlm.nih.gov/geo/), comprising single‐cell transcriptome data from five adjacent OC tissues and six OC tissues. In addition, transcriptomic, somatic mutation, and clinical data for 429 OC samples from the TCGA‐OV project were acquired from the Cancer Genome Atlas (TCGA) database. Samples lacking complete survival information or with survival days < 30 were excluded from both survival analysis and model construction. Expression profiling data for 72 healthy ovarian tissue specimens were retrieved from the Genotype‐Tissue Expression (GTEx) repository. Furthermore, OC genome‐wide association meta‐analysis statistics were sourced from the IEU Open GWAS platform (https://gwas.mrcieu.ac.uk/), encompassing 1218 confirmed OC patients and 198,523 population‐matched control subjects.

The scRNA‐seq dataset GSE184880 was processed following the Seurat pipeline in R. Quality control filtering excluded cells expressing < 200 genes or demonstrating > 20% mitochondrial gene expression. Intersample batch correction was implemented via the Harmony algorithm, while the Top 2000 most variably expressed genes were selected using Seurat’s feature selection module. Dimensionality reduction was achieved through principal component analysis (PCA). Cellular cluster‐defining markers were determined using default thresholds in Seurat’s differential expression analysis module. Cell type annotation was performed leveraging the CellMarker 2.0 repository (http://117.50.127.228/CellMarker/).

scPagwas utilizes a multivariate genetic regression framework to discern phenotype‐associated genes and characterize disease‐relevant cellular clusters through integrative analysis of pathway activity‐transformed single‐cell transcriptomic profiles and GWAS data. The current investigation applied this computational framework to pinpoint critical epithelial cellular subsets in ovarian carcinoma.

BayesPrism is an advanced technology based on a Bayesian model that integrates scRNA‐seq data as a reference profile into bulk RNA‐seq data, facilitating cell type scoring and the inference of posterior gene expression distributions. In this study, the R package “BayesPrism” was used to project cell subtypes identified from scRNA‐seq data in OC onto bulk RNA‐seq data from the TCGA‐OV project, and each epithelial cell subtype was scored.

Limma (Linear Models for Microarray Data) employs generalized linear regression frameworks for transcriptome‐wide differential expression screening [14]. In this investigation, the limma package (v3.40.6) in R was implemented to detect transcriptionally altered genes across experimental cohorts relative to controls. Significance thresholds were defined as Benjamini–Hochberg adjusted *p* < 0.05 combined with absolute log_2_‐transformed fold change exceeding 1. Analytical outcomes were graphically represented through volcano plots and heat maps generated using the R ggplot2 visualization toolkit.

Weighted gene coexpression network analysis (WGCNA) was implemented using the R package “WGCNA” to establish gene coexpression networks. Initially, sample clustering was conducted to identify potential outliers. Subsequently, an automatic network construction algorithm generated the coexpression framework. The “pickSoftThreshold” function determined optimal soft threshold power (*β*), followed by adjacency matrix computation using coexpression similarity measures. Module identification was achieved through hierarchical clustering and dynamic tree cutting algorithms. Module‐trait associations were assessed by correlating gene significance with module membership parameters. Genes within trait‐associated modules were subsequently isolated for downstream investigations.

CSIOCDB (http://csiOCdb.mc.ntu.edu.tw/CSIOCDB.html) is a comprehensive database that integrates gene expression profiles from 3431 human ovarian malignancies, including primary OC, fallopian tube cancer, peritoneal cancer, and secondary ovarian metastatic tumors. This resource also includes data on stromal components and surface epithelium from normal ovarian tissues. Among these, 1868 and 1516 samples have follow‐up data for overall survival (OS) and disease‐free survival (DFS), respectively [15]. This study explored the correlation between target genes and both OS and DFS in OC using the CSIOCDB database.

NetworkAnalyst 3.0 (https://www.networkanalyst.ca) is a powerful online bioinformatics analysis platform specifically designed for gene expression data mining, network construction, and multiomics integration analysis. In this study, the NetworkAnalyst 3.0 database was used to predict the microRNAs (miRNAs) and transcription factors (TFs) that regulate IPO9, and a corresponding regulatory network was constructed.

GeneMANIA (http://genemania.org/) is a powerful and widely used online tool that integrates various bioinformatics data from multiple data sources, including protein–protein interactions (PPIs), gene coexpression, and protein complexes. In this study, the GeneMANIA database was used to explore 20 proteins that interact with IPO9, and a PPI network was constructed.

### 2.13. Statistical Analysis

Analytical methods for intergroup comparisons were selected according to data distribution patterns: Student’s *t*‐test for normally distributed datasets versus the Mann–Whitney *U* test for nonparametric conditions. Multigroup comparisons employed the Kruskal–Wallis nonparametric approach. Bivariate associations were evaluated through Spearman’s rank correlation coefficient analysis. Survival probability estimations utilized the Kaplan–Meier curves with between‐group differences assessed via log‐rank testing. All experiments were performed with at least three biological replicates, and each data point in the figure represents an independent biological replicate. All computational procedures were implemented in R (v4.3.1), with statistical significance thresholds defined as  ^∗^ *p* < 0.05,  ^∗∗^ *p* < 0.01,  ^∗∗∗^ *p* < 0.001, and ns = not significant (*p* ≥ 0.05).

## 3. Results

### 3.1. scRNA‐seq Analysis Combined With scPagwas Identifies Core Epithelial Cells in OC

To gain deeper insights into the immune microenvironment characteristics of ovarian malignancies at single‐cell resolution, we performed scRNA‐seq on datasets obtained from public repositories (Figure 2). Computational analysis revealed 26 distinct cellular clusters across comparative sample groups (Figure 3a), subsequently classified into 10 distinct cellular populations including cytotoxic T lymphocytes (CD8^+^), natural killer cells, phagocytic macrophages, stromal fibroblasts, epithelial components, B lymphocytes, vascular endothelial cells, contractile smooth muscle cells, immunoglobulin‐secreting plasma cells, and helper T cells (CD4^+^) (Figure 3b). Epithelial cells were subsequently isolated for clustering analysis, which led to the identification of 11 distinct epithelial cell subtypes across both groups (Figure 3c,d). scPagwas analysis, integrated with GWAS summary data, was then performed to compute the trait‐relevant score (TRS) for each epithelial cell subtype. Among the 11 epithelial cell types, MALAT1^+^ epithelial cells exhibited significantly higher TRSs than the other subtypes, followed by IGF2^+^ and CENPF^+^ epithelial cells (Figure 3e). Further bootstrap analysis demonstrated a positive correlation between MALAT1^+^ epithelial cells and OC (Figure 3f,g, *p* < 0.05). These results suggest that MALAT1^+^ epithelial cells represent a potential core subpopulation in OC.

Figure 2Single‐cell transcriptome sequencing analysis. (a) Transcriptomic information of each sample. (b) Dendrogram for resolution selection in dimensionality reduction.

**Figure:**
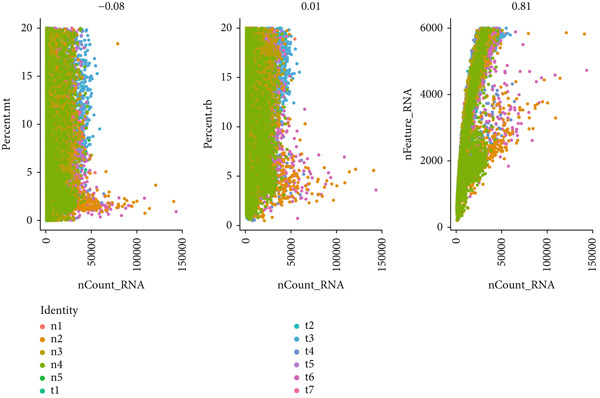
(a)

**Figure:**
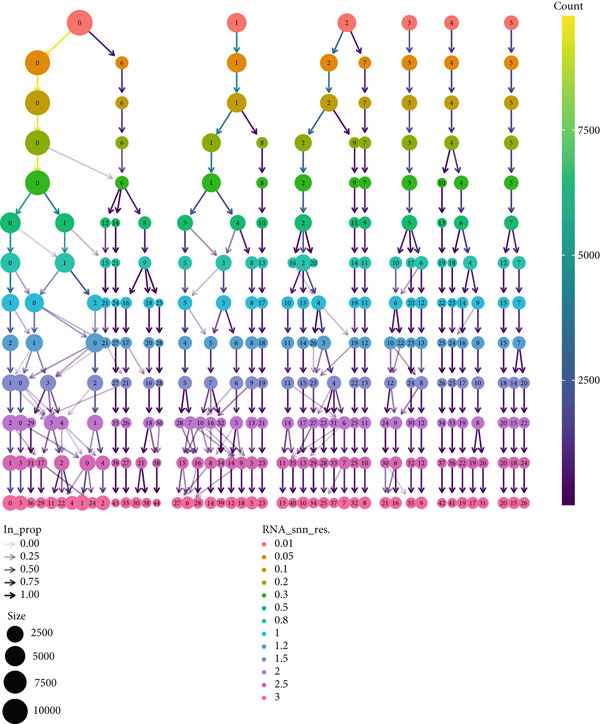
(b)

Figure 3Single‐cell transcriptome sequencing analysis combined with scPagwas analysis identified MALAT1^+^ epithelial cells as the core cell subpopulation in OC. (a) tSNE plot of single‐cell transcriptome clustering and dimensionality reduction analysis. (b) tSNE plot of cell subtype annotation based on marker genes. (c) tSNE plot of reclustering and dimensionality reduction analysis of epithelial cells. (d) tSNE plot of epithelial cell subtype annotation based on marker genes. (e) TRSs of epithelial cell subpopulations by scPagwas analysis. (f) *p* values of bootstrap results for epithelial cell subpopulations in scPagwas analysis. (g) Effect size estimates of epithelial cell subpopulations on OC by scPagwas analysis.

**Figure:**
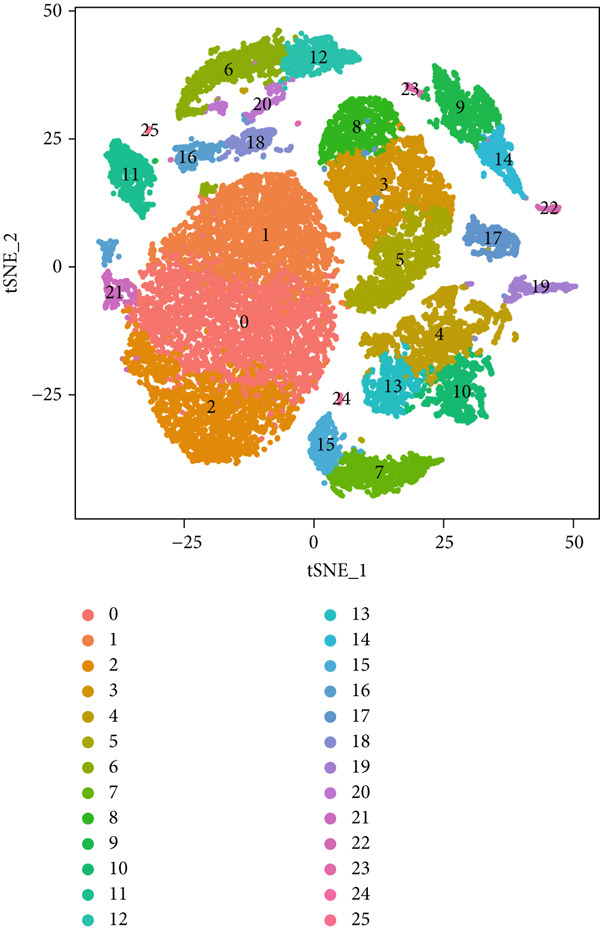
(a)

**Figure:**
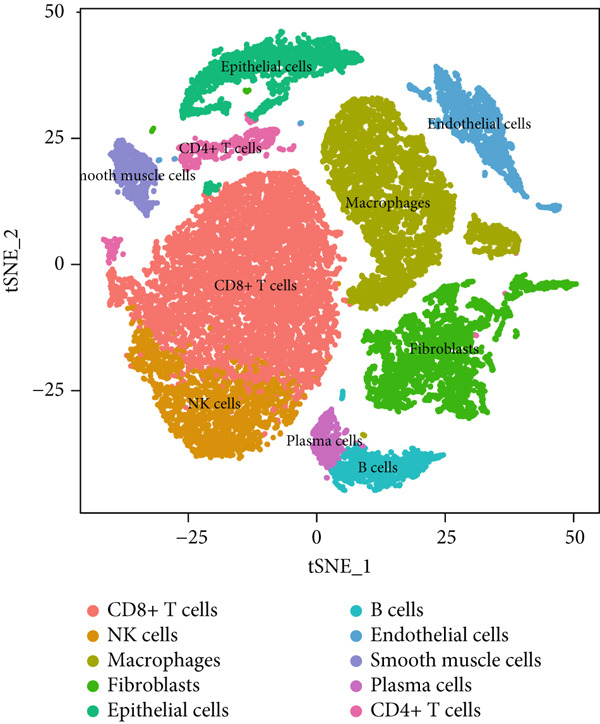
(b)

**Figure:**
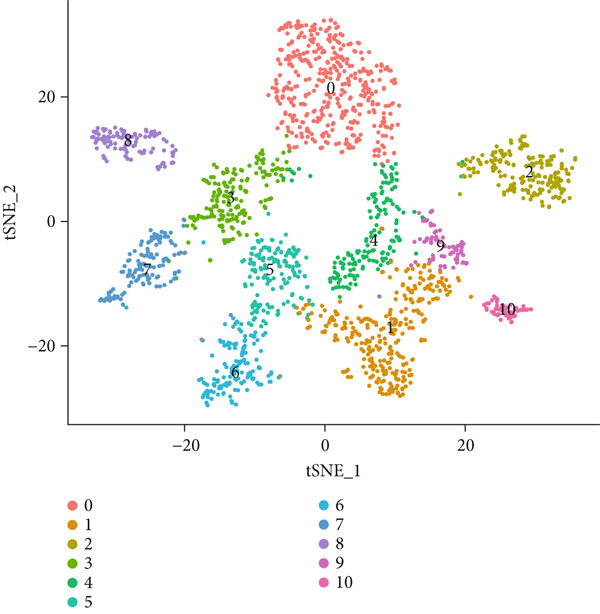
(c)

**Figure:**
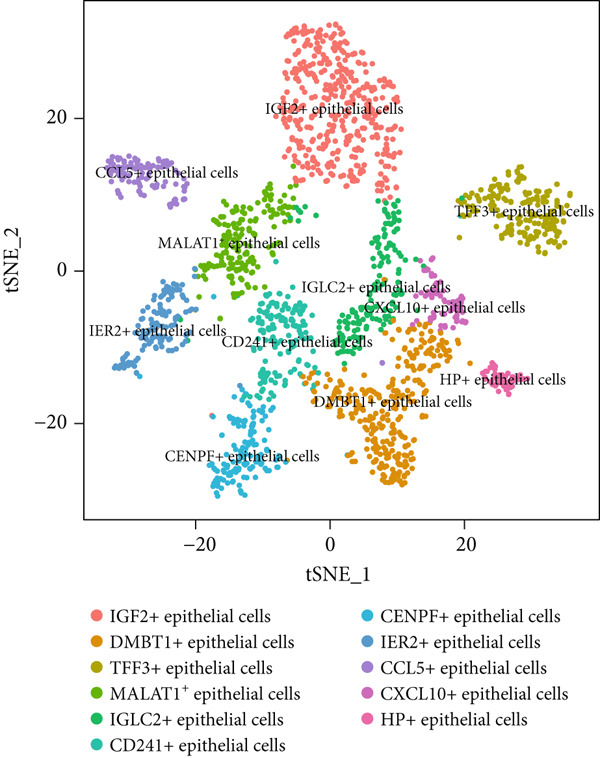
(d)

**Figure:**
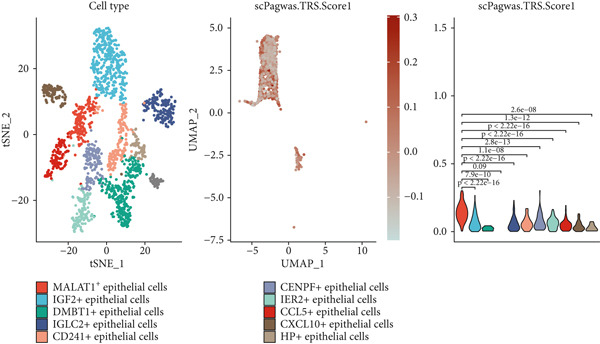
(e)

**Figure:**
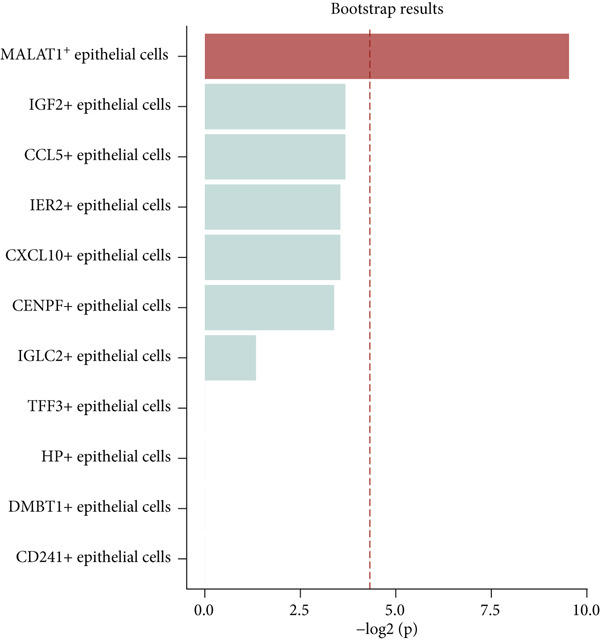
(f)

**Figure:**
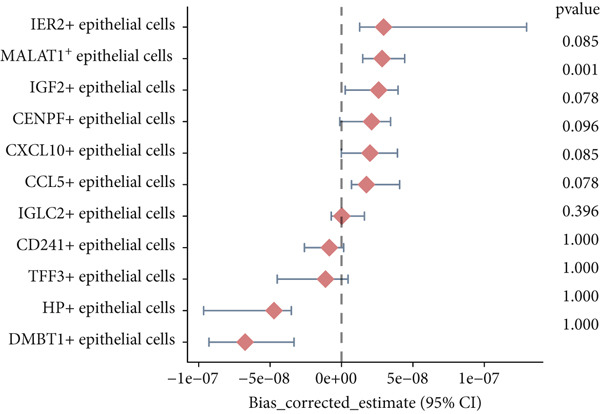
(g)

### 3.2. MALAT1^+^ Epithelial Cells as Core Cells in OC Are Associated With Patient Prognosis

Comparative transcriptomic profiling integrating TCGA‐OV and GTEx ovarian specimens revealed 7644 molecular species exhibiting dysregulation in OC specimens relative to normal controls, comprising 2621 demonstrating elevated expression and 5023 showing reduced levels (Figure 4a). Subsequent computational deconvolution of scRNA‐sequencing data within the TCGA‐OV cohort enabled quantitative assessment of 11 epithelial subpopulations through implementation of the BayesPrism algorithm (Figure 4b), followed by survival correlation assessments. Analytical outcomes identified MALAT1‐positive epithelial populations as demonstrating significant prognostic relevance in OC. Survival curves delineated reduced OS durations for patients displaying elevated MALAT1^+^ epithelial infiltration compared to counterparts with lower cellular prevalence (Figure 4c, *p* < 0.05). Conversely, remaining cellular categories failed to establish statistically meaningful associations with patient survival outcomes (Figure 4c, *p* > 0.05).

Figure 4BayesPrism algorithm. (a) Volcano plot of gene differential analysis between OC tissues in the TCGA‐OV cohort and normal ovarian tissues in the GTEx database. (b) Abundance of epithelial cell subpopulations in the TCGA‐OV cohort evaluated by BayesPrism algorithm. (c) Correlation between the abundance of epithelial cell subpopulations and OS in the TCGA‐OV cohort.

**Figure:**
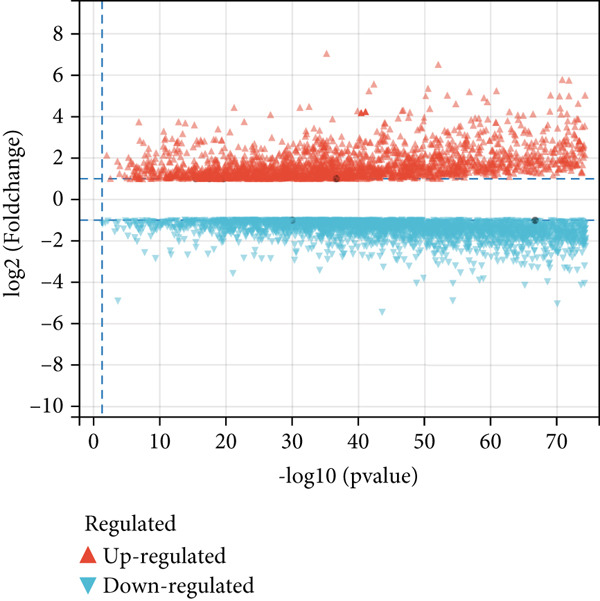
(a)

**Figure:**
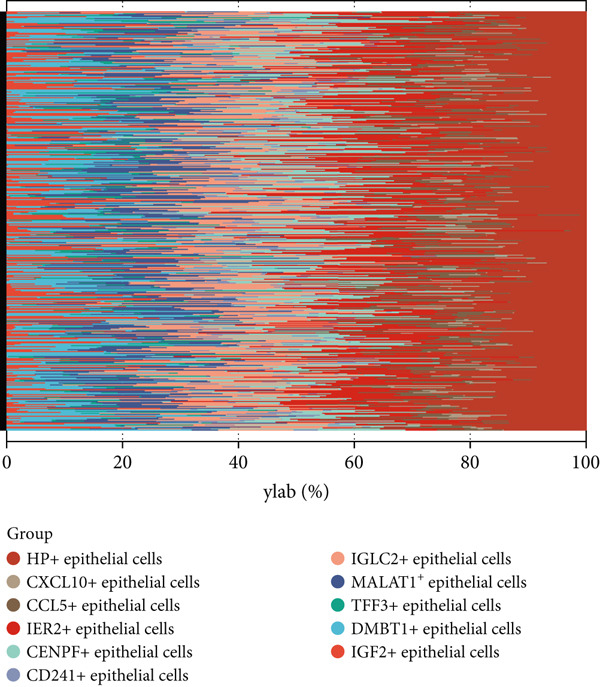
(b)

**Figure:**
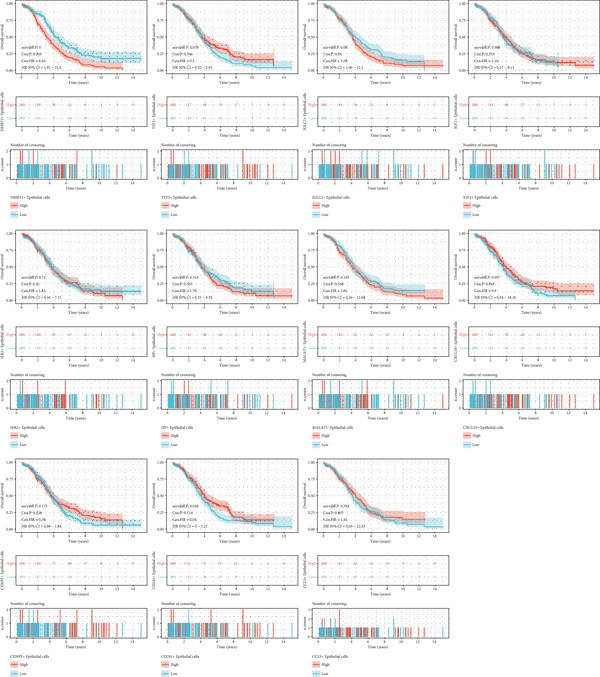
(c)

### 3.3. The Infiltration Level of MALAT1^+^ Epithelial Cells Is Associated With the OC Immune Microenvironment and Immunotherapy Sensitivity

Subsequent differential gene expression profiling was conducted between OC cohorts stratified by MALAT1^+^ epithelial cellular infiltration levels. Transcriptomic comparisons revealed 1414 upregulated transcripts and 1162 downregulated signatures in high‐infiltrating specimens relative to those with lower infiltration (Figure 5a). Enhanced infiltration magnitudes were observed for IGF2^+^, IER2^+^, and CCL5^+^ epithelial subpopulations in high‐infiltrating cohorts, whereas DMBT1^+^, TFF3^+^, IGLC2^+^, CD241^+^, CENPF^+^, CXCL10^+^, and HP^+^ epithelial subtypes demonstrated substantial reductions (Figure 5b). Immune microenvironment characterization via the ESTIMATE computational framework illustrated elevated stromal compartment scores concomitant with diminished immune indices in high‐infiltrating groups, suggesting immunologically quiescent microenvironments (Figure 5b).

Figure 5MALAT1^+^ epithelial cells are associated with the immune microenvironment. (a) Gene differential analysis between high and low MALAT1^+^ epithelial cell groups in the TCGA‐OV cohort. (b) Differences in ESTIMATE scores between high and low MALAT1^+^ epithelial cell groups in the TCGA‐OV cohort.

**Figure:**
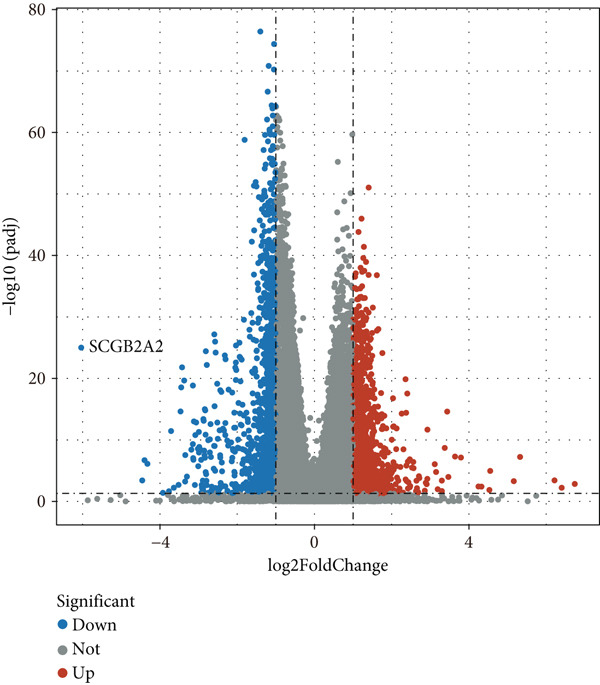
(a)

**Figure:**
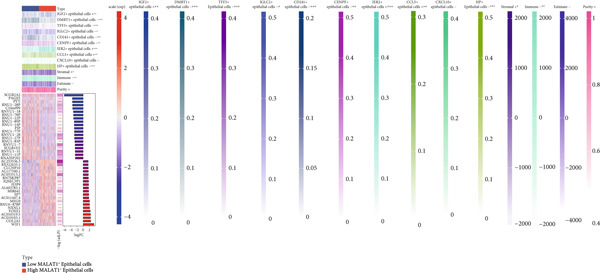
(b)

Given the established connection between MALAT1^+^ epithelial prevalence and OC immune landscapes, we investigated potential immunotherapy response correlations. Comparative analysis demonstrated pronounced increases in CAF infiltration, CD274 (PD‐L1) expression levels, and elevated exclusion/TIDE metrics in high MALAT1 cohorts versus low‐abundance counterparts. These findings collectively indicate immunosuppressive microenvironmental states and potential immunotherapy resistance in MALAT1‐high OC specimens (Figure 6a).

Figure 6MALAT1^+^ epithelial cells are associated with immunotherapy sensitivity and somatic mutations. (a) Differences in immunotherapy sensitivity indicators between high and low MALAT1^+^ epithelial cell groups in the TCGA‐OV cohort. (b, c) Somatic mutation profiles in the TCGA‐OV cohort. (d, e) Differences in somatic mutations between high and low MALAT1^+^ epithelial cell groups in the TCGA‐OV cohort.

**Figure:**
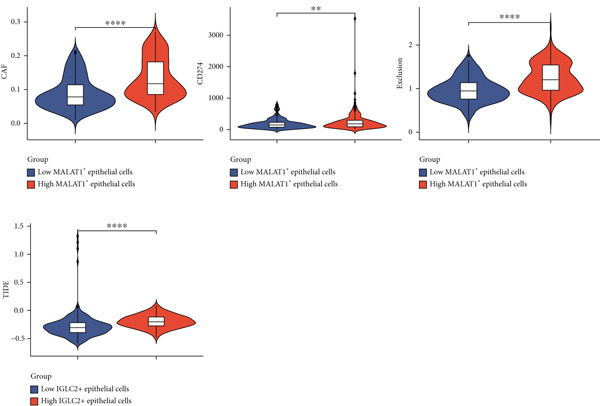
(a)

**Figure:**
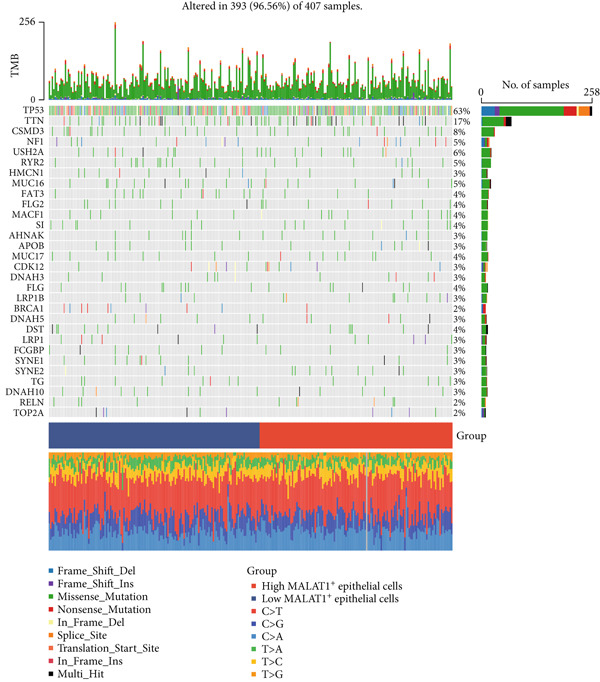
(b)

**Figure:**
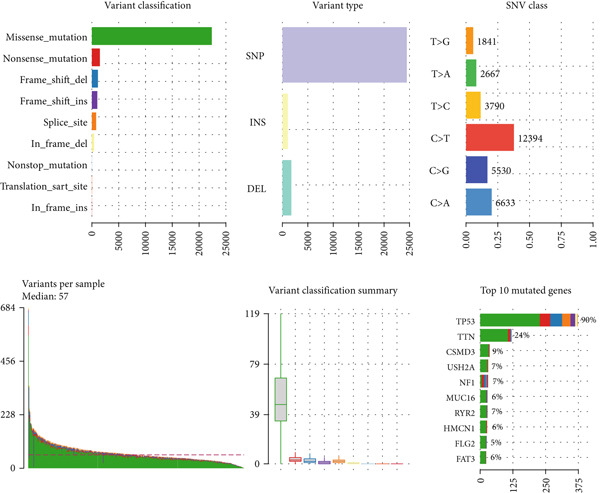
(c)

**Figure:**
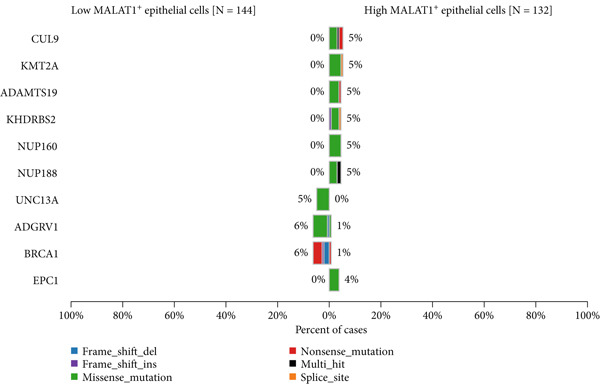
(d)

**Figure:**
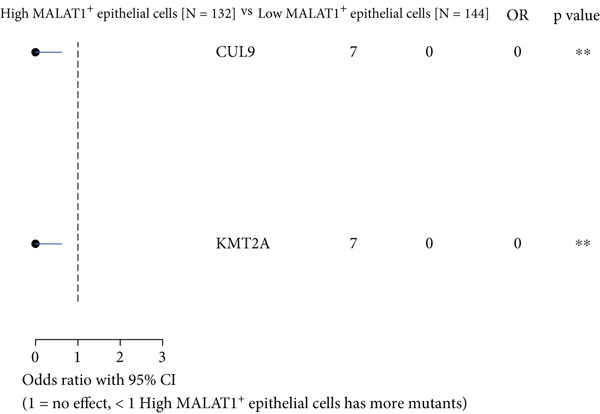
(e)

### 3.4. The Abundance of MALAT1^+^ Epithelial Cells Is Associated With Somatic Mutations in OC

Analysis of somatic mutations across 429 sequenced specimens identified *TP53* genetic alterations as the most prevalent in OC, demonstrating a 63% alteration prevalence predominantly through missense mutations, succeeded by TTN variants (17%) manifesting similar mutational characteristics (Figure 6b,c). Comparative mutational profiling between MALAT1^+^ epithelial cell abundance groups revealed differential mutation patterns: Lower abundance cohorts exhibited increased mutational frequencies in *UNC13A*, *ADGRV1*, and *BRCA1*, whereas elevated abundance groups displayed reduced mutation rates in *CUL9*, *KMT2A*, *AMTS19*, *HDRBS2*, *NUP160*, *NUP188*, and *EPC1*, with particularly notable differentials observed in *CUL9* and *KMT2A* (Figure 6d,e).

### 3.5. Identification of IPO9 as a Core Gene Associated With MALAT1^+^ Epithelial Cells

Considering the potentially significant role of MALAT1^+^ epithelial cells in OC, the present study is aimed at identifying core genes associated with MALAT1^+^ epithelial cells. Under the condition of the optimal soft‐threshold *β* = 7, WGCNA partitioned the genes from the TCGA‐OV dataset into 19 coexpression modules based on gene expression profiles (Figure 7a,b). Correlation analysis revealed that the MEblue (cor = 0.61), MEsalmon (cor = 0.41), MEblack (cor = 0.4), and MEturquoise (cor = 0.4) modules were significantly positively correlated with the MALAT1^+^ epithelial cells score (Figure 7b, *p* < 0.05). Among these, the MEblue module exhibited the strongest correlation and comprised 2511 genes. Intersection of the 2511 MEblue genes, 7644 differentially expressed genes from bulk transcriptome data, and MALAT1^+^ epithelial cell markers from single‐cell transcriptome data identified eight genes: *IGF2BP2*, *HOMER2*, *CHD7*, *PPP1R16A*, *SEMA3F*, *KLHL14*, and *IPO9* (Figure 7c). Survival analysis indicated that high expression levels of *HOMER2*, *IPO9*, and *KLHL14* were associated with poorer OS and DFS, whereas elevated *IGF2BP2* expression correlated with inferior OS and high *SEMA3F* expression correlated with inferior DFS. The remaining molecules did not show a significant association with OC prognosis (Figure 8a). Considering that previous studies have established the association of *HOMER2* and *KLHL14* with OC, whereas *IPO9* has not yet been implicated, subsequent investigations focused on *IPO9*. Results from the NetworkAnalyst tool indicated that the expression of *IPO9* is regulated by TFs including *POU5F1*, *POU3F3*, *POU3F2*, *POU3F1*, *POU2F2*, *POU2F1*, and *CTCF*, as well as miRNAs such as *hsa-miR-1*, *hsa-miR-103*, *hsa-miR-107*, *hsa-miR-150*, *hsa-miR-16*, *hsa-miR-195*, *hsa-miR-206*, *hsa-miR-338-3p*, and *hsa-miR-378* (Figure 8b). PPI analysis showed that the Top 20 proteins interacting with IPO9 were TNPO1, RPS7, CFL1, KPNB1, HTATIP2, IPO5, IPO8, IPO7, NAP1L1, PPP2R1A, PDCD6IP, SLC25A1, MYC, TNPO2, TWF1, IPO11, RANBP17, XPO7, TNPO3, and RPAP1 (Figure 8c).

Figure 7WGCNA identified genes associated with MALAT1^+^ epithelial cells. (a) Dendrogram of WGCNA. (b) Correlation heat map of WGCNA. (c) Intersection of core genes, differentially expressed genes between OC and normal tissues, and MALAT1^+^ epithelial cell marker genes in WGCNA.

**Figure:**
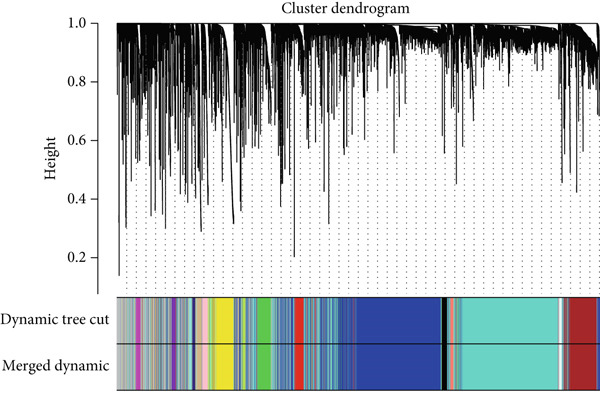
(a)

**Figure:**
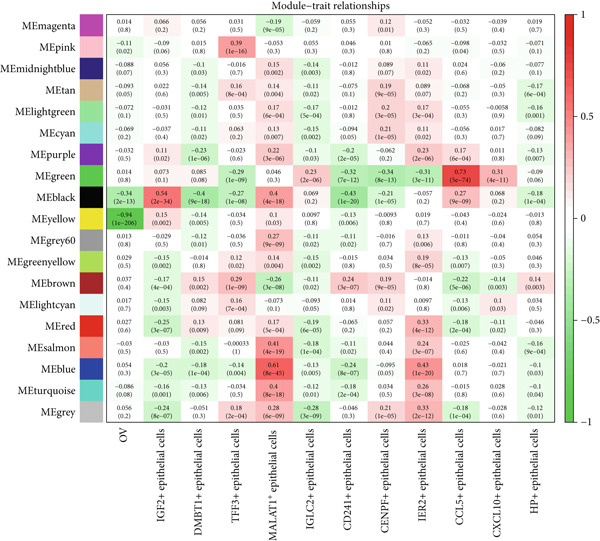
(b)

**Figure:**
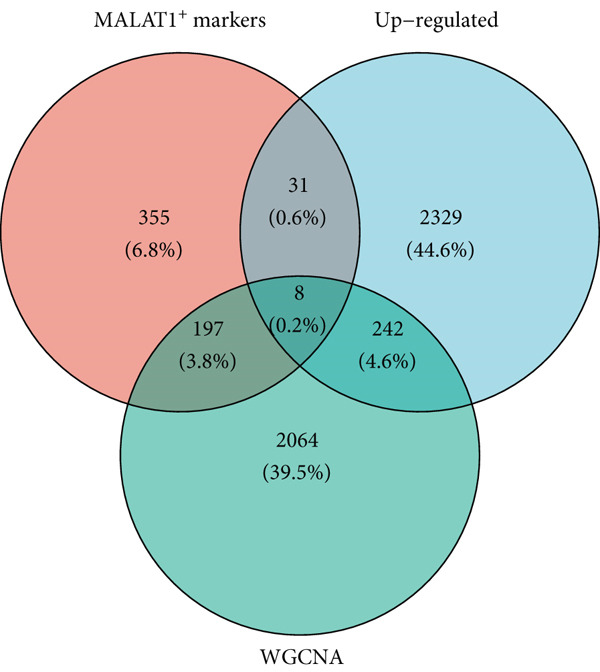
(c)

Figure 8Identification of *IPO9* as a core gene in OC and exploration of its regulatory network. (a) Correlation between MALAT1^+^ epithelial cell–related genes and OS/DFS of OC patients in the CSIOCDB database. (b) Transcription factor–microRNA–*IPO9* regulatory network. (c) Protein–protein interaction network.

**Figure:**
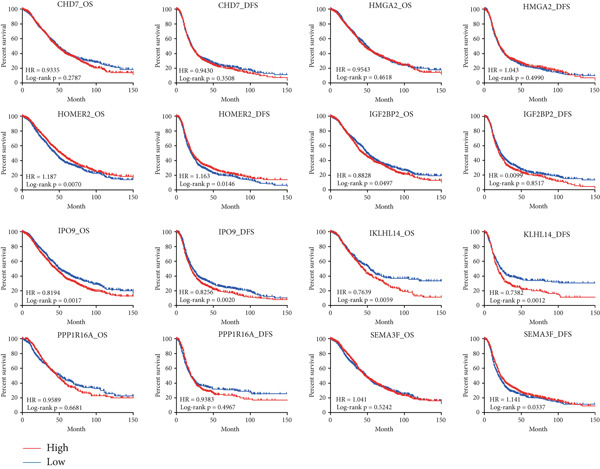
(a)

**Figure:**
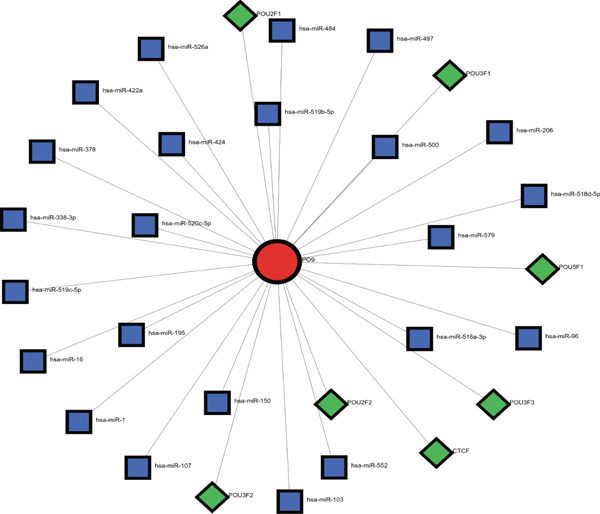
(b)

**Figure:**
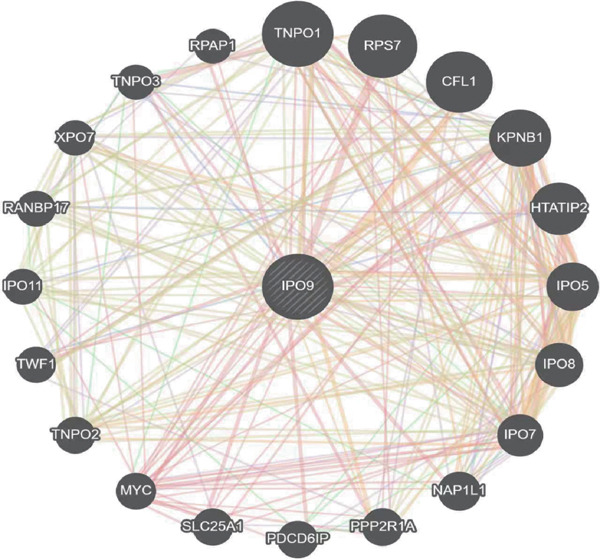
(c)

### 3.6. IPO9 Is Upregulated in OC and Promotes Tumor Progression

Subsequent validation studies were conducted to assess IPO9 expression patterns in clinical specimens. Comparative analysis revealed marked upregulation of IPO9 in OC specimens relative to healthy ovarian counterparts (Figure 9a and Figure S1A). Cellular expression profiling employing qRT‐PCR and immunoblotting techniques was performed across normal ovarian and malignant cell models. Quantitative assessments demonstrated consistently elevated IPO9 transcript and protein levels in SK‐OV‐3 and OVCAR‐8 OC cell lines when benchmarked against the IOSE‐80 normal ovarian reference line (Figure 9b).

Figure 9IPO9 promotes OC progression. (a) qRT‐PCR and Western blot showing differential expression of IPO9 in OC and control tissues. (b) qRT‐PCR and Western blot showing differential expression of IPO9 in normal ovarian epithelial cell line and OC cell lines. (c) EdU assay showing that *IPO9* knockdown inhibits the proliferation of SK‐OV‐3 and OVCAR‐8 cell lines. (d) Transwell assay showing that *IPO9* knockdown inhibits the migration and invasion of SK‐OV‐3 and OVCAR‐8 cell lines. (e) Colony formation assay showing that *IPO9* knockdown inhibits the proliferation of SK‐OV‐3 and OVCAR‐8 cell lines. (f) EdU assay showing that *IPO9* overexpression promotes the proliferation of SK‐OV‐3 and OVCAR‐8 cell lines. (g) Transwell assay showing that *IPO9* overexpression promotes the migration and invasion of SK‐OV‐3 and OVCAR‐8 cell lines. (h) Colony formation assay showing that *IPO9* overexpression promotes the proliferation of SK‐OV‐3 and OVCAR‐8 cell lines. (i) *IPO9* silencing suppresses tumor volume and weight in OC model mice.

**Figure:**
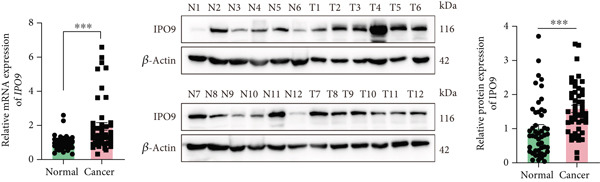
(a)

**Figure:**
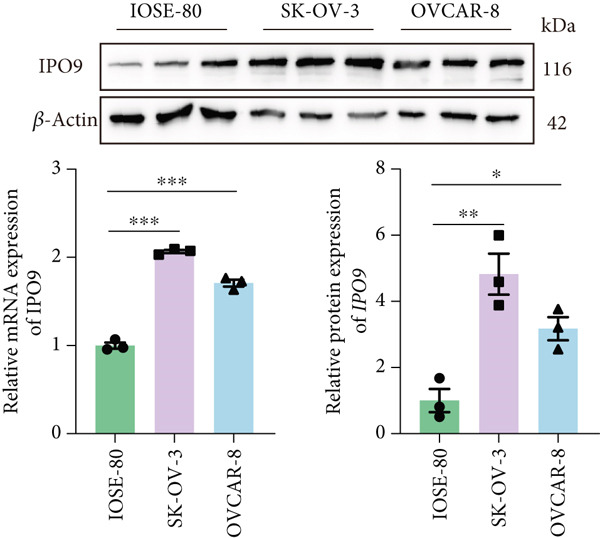
(b)

**Figure:**
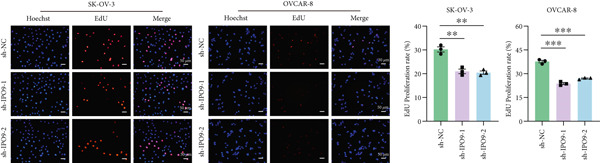
(c)

**Figure:**
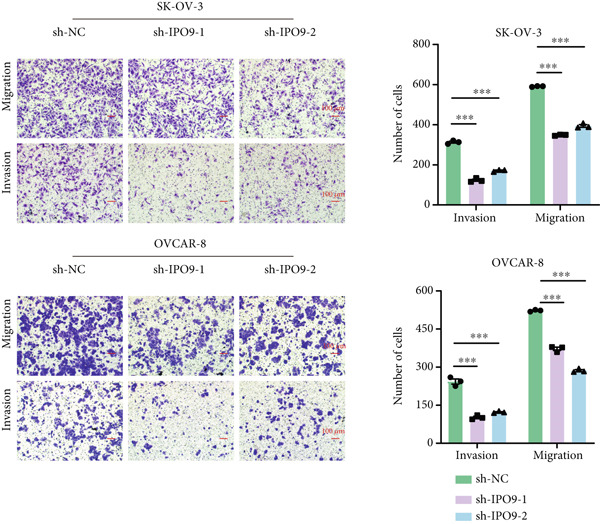
(d)

**Figure:**
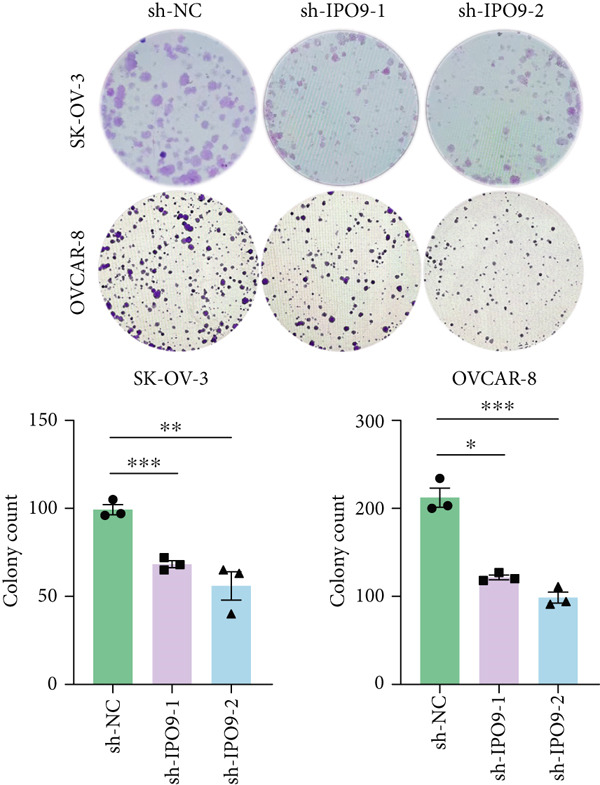
(e)

**Figure:**
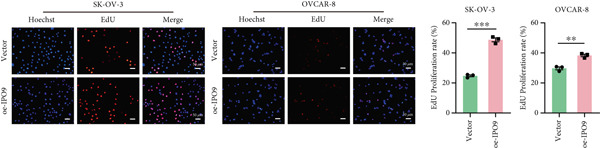
(f)

**Figure:**
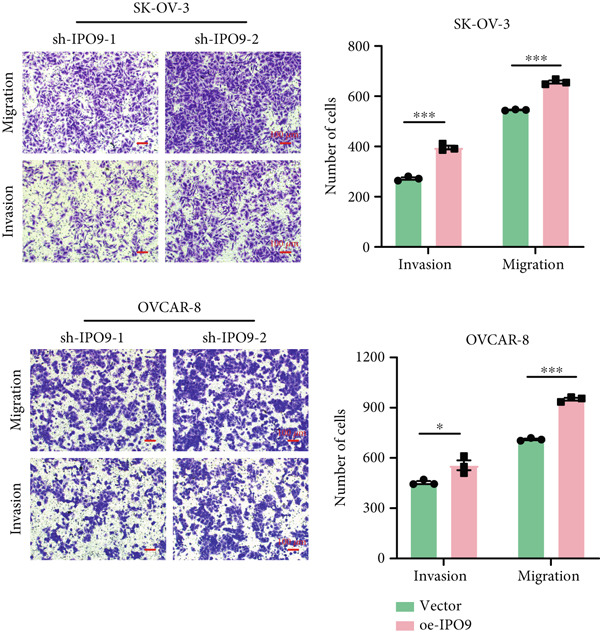
(g)

**Figure:**
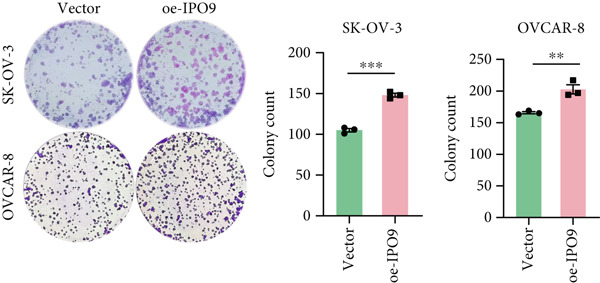
(h)

**Figure:**
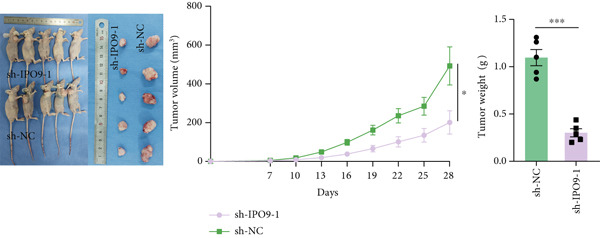
(i)

To investigate IPO9’s functional role in OC pathogenesis, isogenic cell models with *IPO9* suppression and overexpression were established (Figures S1B, S1C, S1D, and S1E). Functional characterization through EdU‐based proliferation assessment, Transwell migration/invasion assays, and clonogenic potential analysis revealed that *IPO9* depletion significantly attenuated malignant cell proliferation capacity, migratory potential, and invasive properties relative to control counterparts (Figures 9c, 9d, and 9e). Conversely, *IPO9* overexpression augmented these oncogenic phenotypes, demonstrating enhanced proliferative activity, increased migratory competence, and elevated invasive capability in OC cellular models (Figures 9f, 9g, and 9h).

To evaluate IPO9’s in vivo functional significance, xenograft models were generated through subcutaneous implantation of SK‐OV‐3 cells with either shNC (control) or sh‐IPO9‐1 constructs into female BALB/c nude mice (*n* = 5 per cohort). Tumor progression was monitored every 3 days with volumetric measurements prior to terminal sacrifice at 4 weeks postimplantation for tumor resection and gravimetric analysis. Experimental observations demonstrated marked reductions in tumor dimensions, weight, and volumetric growth in sh‐IPO9‐1 cohorts relative to control groups (Figure 9i). To verify the SK‐OV‐3‐sh‐IPO9‐1 efficiency, *IPO9* mRNA expression was examined by qRT‐PCR in tumor tissues of mice (Figure S1F).

### 3.7. IPO9 Promotes OC Progression by Inhibiting HMOX1‐Dependent Ferroptosis

Mechanistic investigations into IPO9’s oncogenic role involved generating IPO9‐depleted SK‐OV‐3 cellular models followed by RNA sequencing. Transcriptomic comparisons identified 263 upregulated and 533 downregulated transcripts in *IPO9*‐silenced cells versus controls (Figure 10a,b). Gene Ontology (GO) annotation demonstrated significant enrichment of dysregulated genes in matrix‐related biological processes including vascular morphogenesis, circulatory system development, extracellular matrix restructuring, and neovascularization pathways (Figure 10c). Pathway analysis via KEGG revealed prominent associations with phagosomal processes and ferroptosis regulatory mechanisms (Figure 10d). Ferroptosis is closely associated with OC progression, and HMOX1 induces ferroptosis, thereby promoting cell death [16]. Differential expression analysis revealed that *HMOX1* expression was significantly upregulated following *IPO9* knockdown, suggesting that HMOX1 may serve as a downstream target of IPO9. Western blot analysis further confirmed the upregulation of HMOX1 after *IPO9* knockdown (Figure 11a), supporting this hypothesis. These findings indicate that IPO9 may regulate ferroptosis through the modulation of HMOX1 expression. In SK‐OV‐3 and OVCAR‐8 cell lines, *IPO9* was knocked down concurrently with *HMOX1* downregulation, and the transfection efficiency was verified via Western blot (Figure 11b and Figure S2). Subsequent observations in OC cells demonstrated that *IPO9* knockdown led to significant increases in the levels of ferrous iron, ROS, and MDA (Figures 11c, 11d, and 11e); however, this effect was abolished when HMOX1 was also knocked down, indicating that *IPO9* inhibits HMOX1‐dependent ferroptosis.

Figure 10Transcriptome sequencing indicates that *IPO9* knockdown is associated with ferroptosis. (a) Volcano plot of differentially expressed genes between *IPO9* knockdown and control groups. (b) Heat map of differentially expressed genes between *IPO9* knockdown and control groups. (c) GO enrichment analysis of differentially expressed genes between *IPO9* knockdown and control groups. (d) KEGG enrichment analysis of differentially expressed genes between *IPO9* knockdown and control groups.

**Figure:**
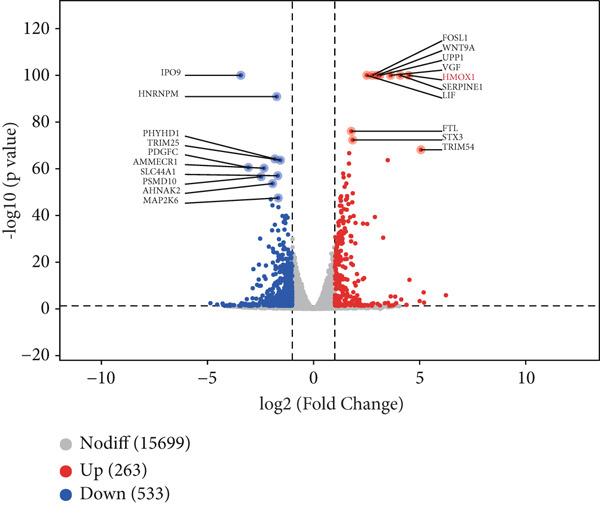
(a)

**Figure:**
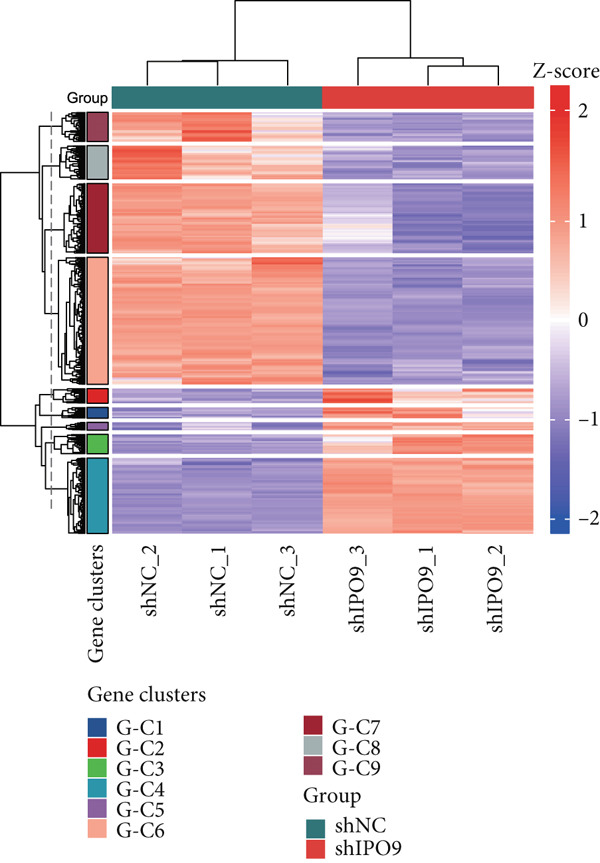
(b)

**Figure:**
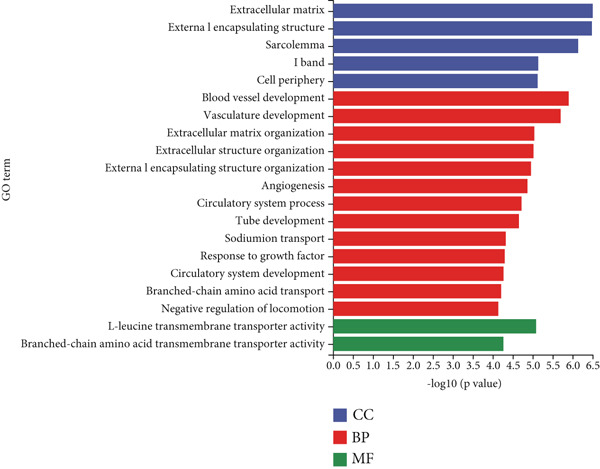
(c)

**Figure:**
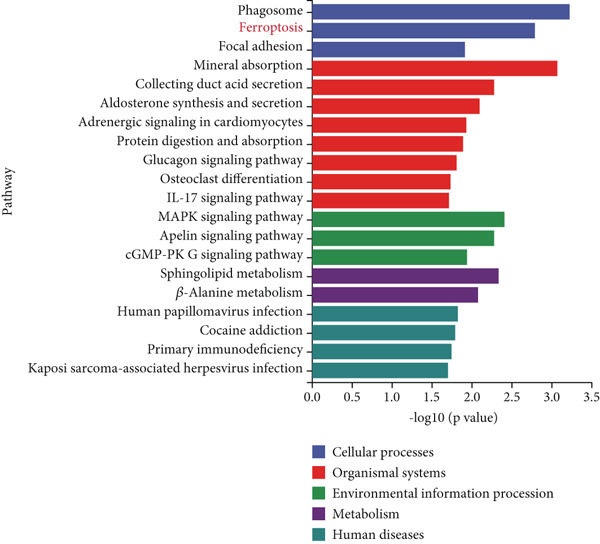
(d)

Figure 11IPO9 inhibits HMOX1‐dependent ferroptosis. (a) Western blot showing that IPO9 knockdown promotes HMOX1 expression in SK‐OV‐3 and OVCAR‐8 cell lines. (b) Western blot analysis: transfection efficiency of rescue experiment. (c) *IPO9* knockdown increases ferrous ion levels in SK‐OV‐3 and OVCAR‐8 cell lines, which is reversed by HMOX1 knockdown. (d) *IPO9* knockdown increases ROS levels in SK‐OV‐3 and OVCAR‐8 cell lines, which is reversed by HMOX1 knockdown. (e) *IPO9* knockdown increases MDA levels in SK‐OV‐3 and OVCAR‐8 cell lines, which is reversed by *HMOX1* knockdown.

**Figure:**
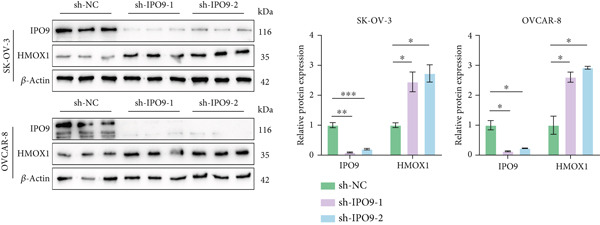
(a)

**Figure:**
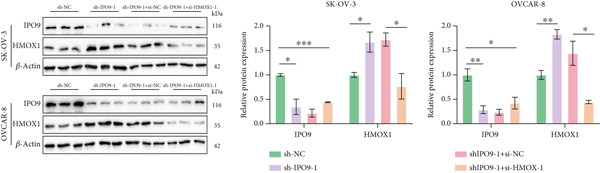
(b)

**Figure:**
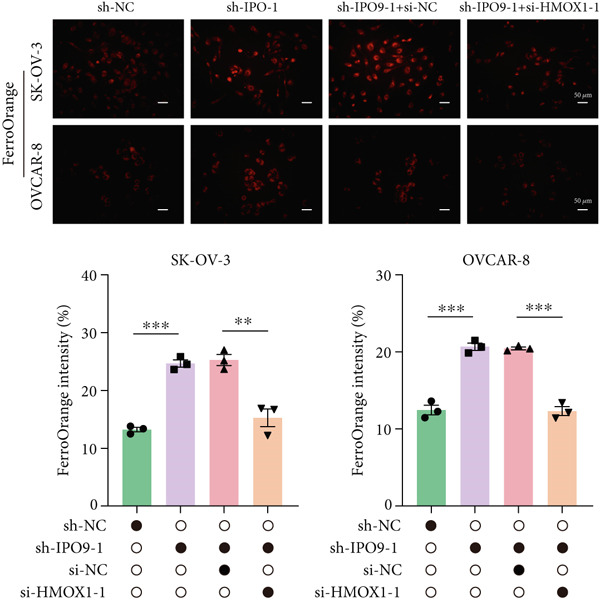
(c)

**Figure:**
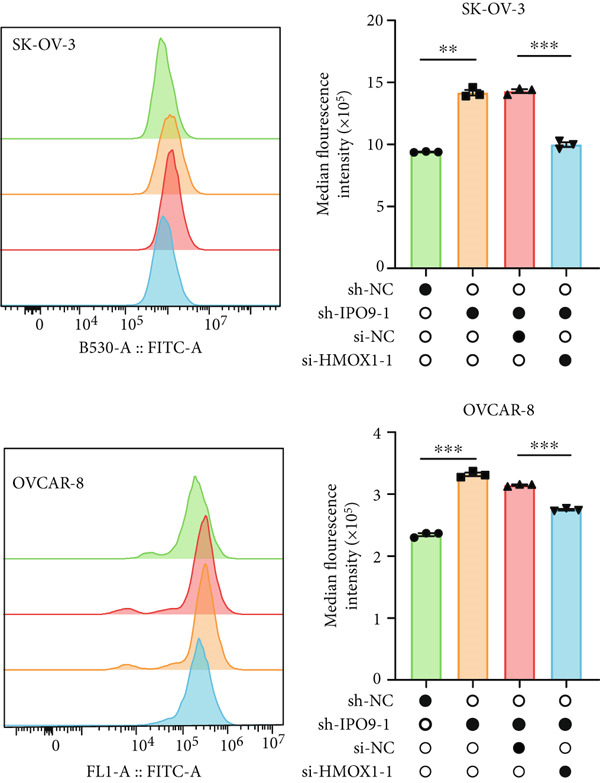
(d)

**Figure:**
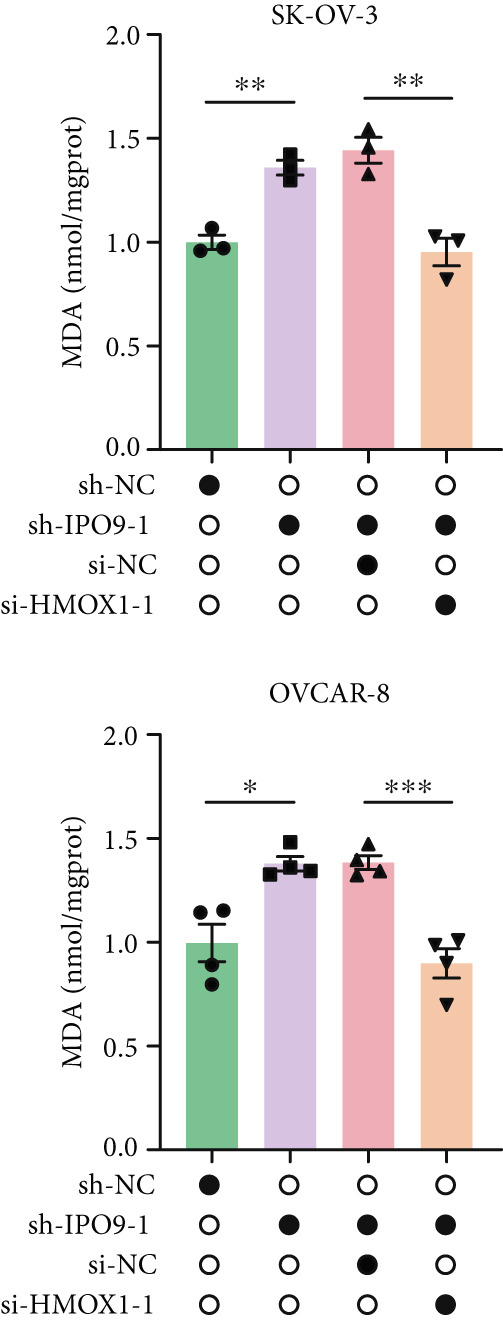
(e)

## 4. Discussion

OC is a malignant tumor that significantly endangers women’s health. Its high mortality rate adversely affects patients’ lives and well‐being, imposes a considerable burden on both patients and their families, and exerts substantial pressure on societal medical resources [1]. Currently, treatment strategies for OC primarily include surgery, chemotherapy, and targeted therapy, among others; however, these approaches remain constrained by high recurrence rates and suboptimal therapeutic efficacy in clinical practice [17]. Accordingly, the present study is aimed at elucidating the pathogenesis of OC and identifying novel therapeutic targets and diagnostic biomarkers. Through systematic bioinformatics analysis, MALAT1^+^ epithelial cells were identified as a critical cellular subpopulation in OC development. Moreover, *IPO9*, a core driver gene within MALAT1^+^ epithelial cells, enhances the survival capacity of these cells by inhibiting HMOX1‐dependent ferroptosis, thereby further promoting OC progression.

OC exhibits substantial multidimensional heterogeneity at the genetic, epigenetic, and phenotypic levels, which profoundly influences tumor biological behavior and therapeutic response. Molecular subtyping studies have demonstrated that increased heterogeneity is strongly associated with poor prognosis. The resultant variability in biomarker expression and treatment response frequently underlies the failure of conventional treatment regimens, underscoring the need for precision therapies based on molecular characteristics to improve clinical outcomes [18]. In view of the high heterogeneity in OC, scRNA‐seq analysis combined with scPagwas identified a critical cellular subpopulation of MALAT1^+^ epithelial cells that drive OC progression. This subpopulation is significantly correlated with OC prognosis and the tumor immune microenvironment. These findings suggest that the abundance of MALAT1^+^ epithelial cells may serve as a potential prognostic indicator, thereby facilitating the development of personalized treatment strategies. The unfavorable prognosis observed in OC patients with a high abundance of MALAT1^+^ epithelial cells may be attributable to the enhanced tumor malignancy associated with elevated MALAT1 expression within this subpopulation. MALAT1 is an lncRNA that has attracted considerable attention in recent years due to its crucial involvement in diverse biological processes and diseases, particularly cancer. MALAT1 plays a critical role in tumorigenesis, metastasis, and the regulation of gene expression and is closely associated with poor prognosis in multiple malignancies. For instance, in osteosarcoma, elevated MALAT1 expression is significantly correlated with clinical stage progression and distant metastasis, indicating its potential as an independent predictor of OS in patients [19]. In prostate cancer, MALAT1 facilitates tumor cell proliferation, migration, and invasion by modulating signaling pathways, including the miR‐140/BIRC6 axis, thus contributing to tumor progression [20]. Mechanistically, MALAT1 often functions as a ceRNA that modulates miRNA activity via a sponge adsorption effect, thereby affecting the expression of target genes. For example, in hepatocellular carcinoma, MALAT1 upregulates SMAD5 expression by sequestering miR‐142‐3p, which promotes epithelial–mesenchymal transition and enhances tumor cell proliferation and invasiveness [21]. This ceRNA mechanism is similarly observed in other cancers, with MALAT1 modulating tumor behavior through interactions with various miRNAs. Furthermore, MALAT1 participates in the regulation of key processes, including cellular autophagy and apoptosis. In colorectal cancer, MALAT1 facilitates tumor cell proliferation while inhibiting apoptosis through autophagy activation, underscoring its pivotal role in tumor cell survival [22]. MALAT1 also exerts an oncogenic effect on OC. Compared with normal ovarian tissues, MALAT1 is markedly overexpressed in OC tissues and cell lines, supporting its role as a potential oncogene in this malignancy [23, 24]. Clinically, elevated MALAT1 expression is closely linked to poor prognosis in OC patients. Studies indicate that high MALAT1 expression is significantly correlated with advanced tumor stage, increased recurrence rates, and reduced OS [25, 26]. At the molecular level, MALAT1 modulates the expression of genes involved in cell cycle regulation and apoptosis. Specifically, MALAT1 downregulation inhibits OC cell proliferation, migration, and invasion, while promoting apoptosis [23, 27]. Moreover, MALAT1 may enhance the metastatic potential of OC by modulating signaling pathways, including Wnt/*β*‐catenin and PI3K/AKT [23, 27, 28]. Downregulation of MALAT1 results in decreased expression of *Dvl2*, *β-catenin*, and *cyclin D1*, accompanied by an increase in *GSK-3β* levels, thereby inhibiting cell proliferation and migration [23]. Furthermore, MALAT1 interacts with miRNAs (e.g., miR‐506) to regulate the expression of target genes associated with OC cell growth [28]. Although the association between MALAT1 and OC has been extensively reported, further validation in additional OC samples is required to confirm the relationship between MALAT1^+^ epithelial cells and OC, thereby determining its universality in OC development and progression and enhancing the reliability of the findings. To investigate molecular determinants underlying MALAT1^+^ epithelial cell prevalence in OC, we implemented WGCNA, differential gene expression profiling, and marker gene identification to pinpoint central regulators. Our integrative approach identified IPO9 as a potential apoptosis‐inhibitory factor safeguarding MALAT1^+^ epithelial populations. Expression pattern analysis revealed IPO9 overexpression in OC tissues correlating with unfavorable clinical outcomes. Emerging evidence implicates IPO9 in oncogenic transformation and neurodegenerative disorders through pathway dysregulation [29, 30]. The investigation provides inaugural evidence of the oncogenic function of IPO9 in OC pathogenesis. Mechanistic interrogation revealed suppression mediated by IPO9 of HMOX1‐regulated ferroptotic pathways, conferring ferroptosis resistance that potentiates malignant progression.

Ferroptosis is a programmed cell death process mediated by iron‐dependent lipid peroxidation that has recently been recognized as a pivotal mechanism in OC management. This form of cell death is distinct from apoptosis and other demise modalities because it is defined by unique biochemical and morphological alterations. For example, it involves the accumulation of ROS and the deterioration of mitochondrial architecture [31]. Abnormalities in iron regulation produce an intracellular surplus of iron that, in turn, amplifies oxidative stress and precipitates the onset of ferroptosis [32]. Enhancing the induction of ferroptosis can elevate the chemosensitivity of OC cells. For example, experimental evidence indicates that iron‐binding agents, by lowering cellular iron levels, render OC cells more vulnerable to platinum‐based treatments while concurrently hindering DNA repair mechanisms [33]. This finding holds considerable importance for therapeutic strategies, as resistance to platinum compounds is a prevalent issue among OC patients that frequently culminates in treatment failure. By facilitating ferroptosis, such resistance may be bypassed, potentially leading to improved clinical responses. Moreover, the initiation of ferroptosis appears to be intertwined with distinct signaling cascades governing tumor progression. Notably, the transcriptional regulation of genes linked to lipid metabolism and antioxidant defense can modulate the propensity of OC cells to undergo ferroptosis [32]. Interactions between ferroptotic processes and the tumor microenvironment, particularly the cross‐talk with immune cells, further contribute to OC therapy [34]. Consequently, ferroptosis has emerged as a central mechanism in restraining OC by promoting oxidative stress, modulating iron balance, and enhancing chemotherapy effectiveness. HMOX1, which influences cellular iron equilibrium and apoptotic pathways, is recognized as a proferroptotic gene whose overexpression is associated with the induction of ferroptosis. By catalyzing heme degradation and liberating free iron, HMOX1 facilitates iron‐dependent lipid peroxidation, thereby initiating ferroptotic cell death. This mechanism not only highlights the therapeutic promise of ferroptosis in oncology but also furnishes a conceptual framework for designing interventions that target HMOX1. Manipulation of HMOX1 expression or function may thus refine strategies aimed at inducing ferroptosis, ultimately improving treatment efficacy against OC [35]. The present investigation demonstrated that IPO9, an emerging factor implicated in OC progression, suppresses HMOX1‐mediated ferroptosis. In light of preceding studies and the current observations, the hypothesis is posited that MALAT1^+^ epithelial cells are instrumental in both the onset and advancement of OC and that IPO9 confers resistance to ferroptosis by inhibiting HMOX1‐dependent cell death in MALAT1^+^ epithelial cells, thus fostering cell survival and facilitating tumor progression.

## 5. Conclusions

Utilizing multiple bioinformatics analysis methods, the current study examined the immune microenvironment of OC at the single‐cell level and identified MALAT1^+^ epithelial cells as a candidate core subpopulation in OC. Moreover, IPO9 facilitates OC progression through inhibition of the HMOX1‐dependent ferroptosis process. These observations yield valuable insights into the pathogenesis of OC and may reveal novel targets and therapeutic strategies for its diagnosis and treatment.

**Nomenclature**
DFS: disease‐free survival
GEO: Gene Expression Omnibus
GTEx: Genotype‐Tissue Expression
GWAS: genome‐wide association study
MDA: malondialdehyde
miRNAs: microRNAs
OC: ovarian cancer
OS: overall survival
PPI: protein–protein interaction
qRT‐PCR: quantitative real‐time polymerase chain reaction
ROS: reactive oxygen species
scRNA‐seq: single‐cell RNA sequencing
TCGA: the Cancer Genome Atlas
TFs: transcription factors
WGCNA: weighted gene coexpression network analysis

## Ethics Statement

The use of human tissues for our experiments received approval from the Ethics Committee of the Second Affiliated Hospital of Harbin Medical University (Harbin, China), with informed consent obtained from all participants (Approval No. Ky2020‐060). Furthermore, the Animal Ethics Committee of the Second Affiliated Hospital of Harbin Medical University (Harbin, China) approved all procedures involving animals (Approval No. YJSDW2023‐108). All procedures involving human subjects were conducted in accordance with the Declaration of Helsinki and the ethical guidelines of the World Medical Association. Animal experiments were performed in compliance with the Guide for the Care and Use of Laboratory Animals (National Research Council) and the institutional animal welfare regulations.

## Consent

The authors have nothing to report.

## Conflicts of Interest

The authors declare no conflicts of interest.

## Author Contributions

Conceptualization: Peiling Li and Yimei Meng. Funding acquisition: Peiling Li. Supervision: Peiling Li. Visualization: Yimei Meng. Writing—original draft: Yimei Meng. Writing—review and editing: Peiling Li.

## Funding

The study was funded by the National Natural Science Foundation of China (10.13039/501100001809, No. 82072864).

## Supporting information


**Supporting Information** Additional supporting information can be found online in the Supporting Information section. Figure S1. Validation of the efficiency of IPO9. (A) Western blot showing differential expression of IPO9 in OC and control tissues. (B, C) qRT‐PCR and Western blot were used to detect the knockdown efficiency of IPO9 in SK‐OV‐3 and OVCAR‐8 cell lines. (D, E) qRT‐PCR and Western blot were used to detect the overexpression efficiency of IPO9 in SK‐OV‐3 and OVCAR‐8 cell lines. (F) qRT‐PCR was used to detect the expression efficiency of IPO9 in xenograft tumor samples. Figure S2. Validation of the knockdown efficiency with HMOX1 in SK‐OV‐3 and OVCAR‐8 cell lines. (A, B) qRT‐PCR and Western blot were used to detect the knockdown efficiency of HMOX1 in SK‐OV‐3 and OVCAR‐8 cell lines. Table S1. The clinical characteristics of the 48 OC patients enrolled in this study, including age distribution, histological subtypes, FIGO staging, tumor size, and lymph node metastasis status, which provides essential clinical context. Table S2. Lists of the primer sequences used in qRT‐PCR experiments, including primers for the internal reference gene 18s and target genes IPO9 and HMOX1. Table S3. The sequences of two HMOX1‐specific small interfering RNAs (si‐HMOX1‐1 and si‐HMOX1‐2) utilized for endogenous HMOX1 silencing in functional validation assays.

## Data Availability

The data that support the findings of this study are available from the corresponding author upon reasonable request.
