# Supplementary material for: IPO9 Promotes Ovarian Cancer Progression by Suppressing HMOX1‐Dependent Ferroptosis
Source: Hum Mutat. 2026 Jan 21;2026:8545131. doi: 10.1155/humu/8545131 (PMC12824451; doi:10.1155/humu/8545131)
Supplement: Supplementary file 1 — Supporting Information Additional supporting information can be found online in the Supporting Information section. Figure S1. Validation of the efficiency of IPO9. (A) Western blot showing differential expression of IPO9 in OC and control tissues. (B, C) qRT‐PCR and Western blot were used to detect the knockdown efficiency of IPO9 in SK‐OV‐3 and OVCAR‐8 cell lines. (D, E) qRT‐PCR and Western blot were used to detect the overexpression efficiency of IPO9 in SK‐OV‐3 and OVCAR‐8 cell lines. (F) qRT‐PCR was used to detect the expression efficiency of IPO9 in xenograft tumor samples. Figure S2. Validation of the knockdown efficiency with HMOX1 in SK‐OV‐3 and OVCAR‐8 cell lines. (A, B) qRT‐PCR and Western blot were used to detect the knockdown efficiency of HMOX1 in SK‐OV‐3 and OVCAR‐8 cell lines. Table S1. The clinical characteristics of the 48 OC patients enrolled in this study, including age distribution, histological subtypes, FIGO staging, tumor size, and lymph node metastasis status, which provides essential clinical context. Table S2. Lists of the primer sequences used in qRT‐PCR experiments, including primers for the internal reference gene 18s and target genes IPO9 and HMOX1. Table S3. The sequences of two HMOX1‐specific small interfering RNAs (si‐HMOX1‐1 and si‐HMOX1‐2) utilized for endogenous HMOX1 silencing in functional validation assays. [file HUMU-2026-8545131-s001.docx]

Supplementary Material

# Supplementary Figures and Tables

## Supplementary Figures


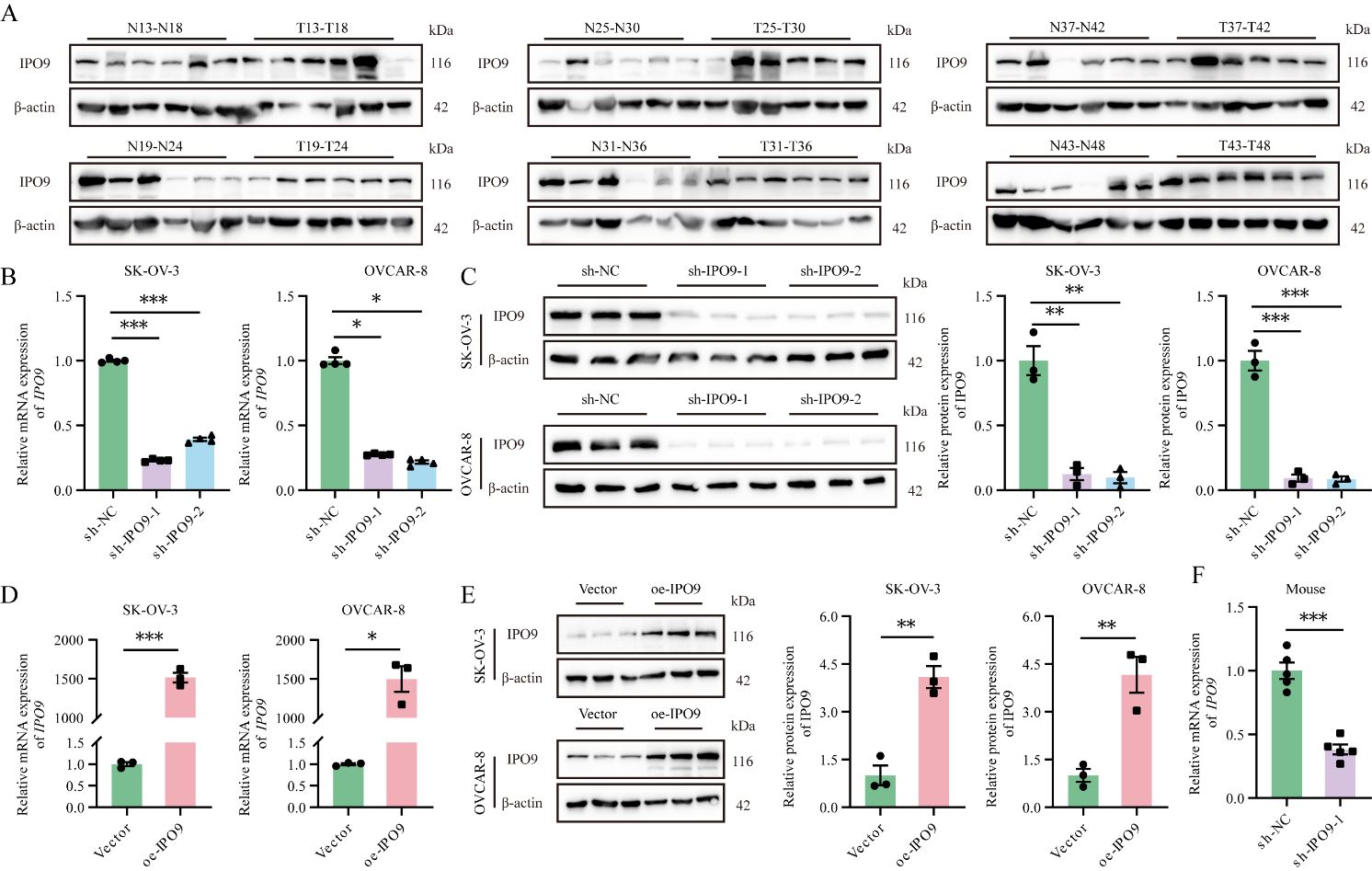


**Supplementary Figure 1.** Validation of the efficiency of IPO9. A. Western blot showing differential expression of IPO9 in OC and control tissues. B-C. RT-qPCR and Western blot were used to detect the knockdown efficiency of IPO9 in SK-OV-3 and OVCAR-8 cell lines. D-E. RT-qPCR and Western blot were used to detect the overexpression efficiency of IPO9 in SK-OV-3 and OVCAR-8 cell lines. F. RT-qPCR was used to detect the expression efficiency of *IPO9* in xenograft tumor samples.


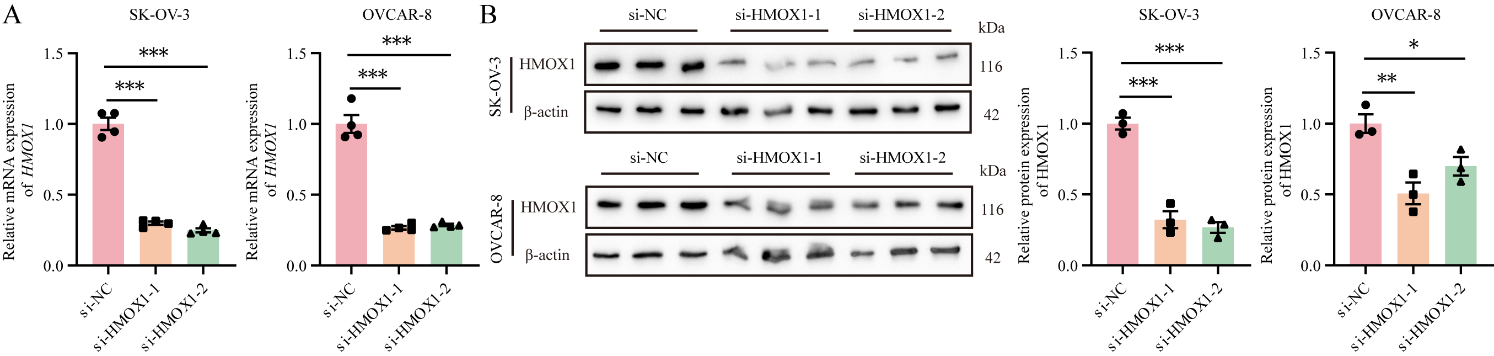


**Supplementary Figure 2.** Validation of the knockdown efficiency with HMOX1 in SK-OV-3 and OVCAR-8 cell lines. A-B. RT-qPCR and Western blot were used to detect the knockdown efficiency of HMOX1 in SK-OV-3 and OVCAR-8 cell lines.

## Supplementary Tables

**Supplementary Table 1.** The clinical characteristics of OC patients.

| Characteristics | Number | Percentage |
| --- | --- | --- |
| n | 48 |  |
| Age |  |  |
| <＝60 | 36 | 75% |
| > 60 | 12 | 25% |
| Histology type |  |  |
| Serous | 30 | 62.5% |
| Mucinous | 10 | 20.8% |
| Endometrioid | 7 | 14.6% |
| Clean cell | 1 | 2.1% |
| FIGO Stage |  |  |
| Ⅰ+Ⅱ | 20 | 41.7% |
| Ⅲ+Ⅳ | 28 | 58.3% |
| Tumor size |  |  |
| <＝10 cm | 22 | 45.8% |
| > 10 cm | 26 | 54.2% |
| Lymph node metastasis |  |  |
| Positive | 38 | 79.2% |
| Negative | 10 | 20.8% |

**Supplementary Table 2.** Primer information used in this study.

| *18s*（F） | ATCCTCAGTGAGTTCTCCCG |
| --- | --- |
| *18s*（R） | CTTTGCCATCACTGCCATTA |
| *IPO9*（F） | AGGAGGATTACTACGAGGATGATGAG |
| *IPO9*（R） | TGAGCAAACTGGCAGAGGAAATC |
| *HMOX1*（F） | AAGACTGCGTTCCTGCTCAAC |
| *HMOX2*（R） | AAAGCCCTACAGCAACTGTCG |

**Supplementary Table 3.** siRNA sequences of HMOX1.

| *si-HMOX1-1* | AGACUGCGUUCCUGCUCAATTUUGAGCAGGAACGCAGUCUTT |
| --- | --- |
| *si-HMOX1-2* | CAGAAGAGCUGCACCGCAATTUUGCGGUGCAGCUCUUCUGTT |
